# The impact of behavioural risk factors on communicable diseases: a systematic review of reviews

**DOI:** 10.1186/s12889-021-12148-y

**Published:** 2021-11-17

**Authors:** Sara Wood, Sophie E. Harrison, Natasha Judd, Mark A. Bellis, Karen Hughes, Andrew Jones

**Affiliations:** 1 Policy and International Health, World Health Organization Collaborating Centre on Investment for Health and Well-being, Public Health Wales, Wrexham, UK; 2 Public Health Collaborating Unit, School of Medical and Health Sciences, Bangor University, Wrexham, UK; 3 Institute for Applied Human Physiology, School of Human and Behavioural Sciences, Bangor University, Bangor, UK; 4 Health Protection and Screening Services, Public Health Wales, Cardiff, UK

**Keywords:** Infectious disease, COVID-19, Risk factors, Obesity, Smoking, Drug use, Alcohol, Prevention, Pandemic, Resilience

## Abstract

**Background:**

The coronavirus (COVID-19) pandemic has highlighted that individuals with behavioural risk factors commonly associated with non-communicable diseases (NCDs), such as smoking, harmful alcohol use, obesity, and physical inactivity, are more likely to experience severe symptoms from COVID-19. These risk factors have been shown to increase the risk of NCDs, but less is known about their broader influence on communicable diseases. Taking a wide focus on a range of common communicable diseases, this review aimed to synthesise research examining the impact of behavioural risk factors commonly associated with NCDs on risks of contracting, or having more severe outcomes from, communicable diseases.

**Methods:**

Literature searches identified systematic reviews and meta-analyses that examined the association between behavioural risk factors (alcohol, smoking, illicit drug use, physical inactivity, obesity and poor diet) and the contraction/severity of common communicable diseases, including infection or associated pathogens. An a priori, prospectively registered protocol was followed (PROSPERO; registration number CRD42020223890).

**Results:**

Fifty-three systematic reviews were included, of which 36 were also meta-analyses. Reviews focused on: tuberculosis, human immunodeficiency virus, hepatitis C virus, hepatitis B virus, invasive bacterial diseases, pneumonia, influenza, and COVID-19. Twenty-one reviews examined the association between behavioural risk factors and communicable disease contraction and 35 examined their association with communicable disease outcomes (three examined their association with both contraction and outcomes). Fifty out of 53 reviews (94%) concluded that at least one of the behavioural risk factors studied increased the risk of contracting or experiencing worse health outcomes from a communicable disease. Across all reviews, effect sizes, where calculated, ranged from 0.83 to 8.22.

**Conclusions:**

Behavioural risk factors play a significant role in the risk of contracting and experiencing more severe outcomes from communicable diseases. Prevention of communicable diseases is likely to be most successful if it involves the prevention of behavioural risk factors commonly associated with NCDs. These findings are important for understanding risks associated with communicable disease, and timely, given the COVID-19 pandemic and the need for improvements in future pandemic preparedness. Addressing behavioural risk factors should be an important part of work to build resilience against any emerging and future epidemics and pandemics.

**Supplementary Information:**

The online version contains supplementary material available at 10.1186/s12889-021-12148-y.

## Background

The recent coronavirus (COVID-19) pandemic has highlighted that individuals with potentially modifiable behavioural risk factors that are commonly associated with non-communicable diseases (NCDs), such as smoking, harmful alcohol use, obesity and physical inactivity, are more likely to experience severe symptoms from COVID-19 infection [1], resulting in greater risk of hospitalisation [2]. With these behavioural risk factors often having higher prevalence in the poorest communities, COVID-19 has disproportionately impacted those already suffering the greatest risks of ill health, thereby widening health and social inequalities [3]. Indeed, due to its associations with existing health and social risk factors, COVID-19 has been referred to as a syndemic; one in which existing health and social challenges increase an individual’s susceptibility to disease [4]. However, whilst addressing behavioural risk factors is routinely considered in the prevention of NCDs, their role in the contraction of communicable disease, and severity of symptoms in those who are infected, has had a lower public health prominence.

Many modifiable behavioural risk factors are highly prevalent among adults and adolescents in both higher (HICs) and lower and middle income countries (LMICs) [5, 6], with levels increasing in many LMICs (e.g. obesity, alcohol) [7, 8]. As a result, NCDs, such as cancer, respiratory disease and cardiovascular disease are the highest cause of mortality and morbidity in HICs and account for a rapidly increasing proportion of both in LMICs [9]. Across countries globally, the burden of NCDs has been found to correlate with levels of COVID-19 cases and deaths [10].

With both international commerce and tourism connecting populations globally, it is highly likely that COVID-19 is only one in a series of existing and emerging infectious diseases likely to impact, to different extents, health and well-being on a global scale [11]. Although the exact nature or source of any future epidemic or pandemic threat is speculative, behavioural risk factors have also been found to increase the risk of infection and subsequent poorer outcomes across a range of other communicable diseases [12–14]. Understanding which factors may increase or reduce risk of contraction and severity of disease can provide important intelligence, both in increasing a population’s resilience to infectious disease, and in identifying which communities and individuals may be most at risk from the spread of different types of disease. Although previous research has explored links between behavioural risk factors and individual communicable diseases, few studies have synthesised information across a wider range of communicable diseases and their relationships with behavioural risks. Indeed, such relationships may elucidate how future pandemics threats will exploit behavioural risk factors.

Intending to explore whether communicable diseases and NCDs share a common set of behavioural risk factors, the aim of this review was to provide a synthesis of existing research examining the impact of behavioural risk factors commonly associated with NCDs on the risk of people (adults or children) contracting, or experiencing more severe outcomes from, common communicable diseases. With the breadth of communicable diseases requiring limitation, the focus of this review was on diseases common to high income countries. With an intentionally wide focus on a range of communicable diseases, the review focused specifically on systematic reviews and meta-analyses, clarifying existing knowledge and highlighting gaps in evidence to inform priority areas for future research.

## Methods

This review was carried out in adherence to the Preferred Reporting Items for Systematic Reviews and Meta-Analyses (PRISMA) guidelines. An a priori protocol was followed and prospectively registered at the National Institute for Health Research international prospective register of systematic reviews (PROSPERO) (registration number CRD42020223890). The focus of this review was limited to behavioural risk factors and communicable diseases common in HICs, regardless of the geographical location of the review. Those more specific to LMICs or certain regions of the world (e.g. tropical diseases) were considered best examined in a separate study.

### Search strategy

A systematic search was performed across multiple databases through ProQuest covering the 10-year period 28th October 2010 to 28th October 2020. Preliminary scans of the literature reviews and discussion between members of the research team were used to aid selection of common behavioural risk factors and communicable diseases. Thus, alcohol use, smoking, physical inactivity, obesity, illicit drug use and poor diet were chosen as behavioural risk factors, covering some of the most common behavioural contributors to NCDs [15]. The same process identified: Tuberculosis (TB), acquired immune deficiency syndrome (AIDS), human immunodeficiency virus (HIV), viral hepatitis, COVID-19, severe acute respiratory syndrome (SARS), middle-east respiratory syndrome (MERS), pneumonia, influenza, and meningitis as communicable diseases (including infections and pathogens) feasible for review. These communicable diseases were broadly consistent with some of the most prevalent disease/infection categories reported in the global burden of disease study for HICs (excluding those categories predominantly affecting specific groups (e.g. maternal, neonatal) or where a component may be associated with non-infectious causes (e.g. diarrheal) [9]) and with previous outbreaks or epidemics involving HICs [16]. In addition, it was intended to include diseases that arose from both bacterial and viral pathogens, with a range of transmission types, e.g. airborne, droplet, fomite, blood-borne and contact. Combinations of search terms were developed based on these key risk factors and diseases. Search results were restricted to English language and peer reviewed systematic reviews and meta-analyses. Whilst this strategy restricted literature to that which qualified for inclusion in systematic reviews, it allowed for the inclusion of multiple behavioural risk factors and communicable diseases at the same time. The search was restricted to a 10-year period, allowing coverage of a broad range of behavioural risk factors and communicable diseases, yet limiting the literature to a manageable volume. The full search strategy is available in Supplementary file 1. Searches also included poor housing conditions as a risk factor given the impact of housing conditions on respiratory disease [17], but this study focuses specifically on behavioural risk factors.

### Study selection and eligibility criteria

To identify eligible studies, the titles and abstracts of studies retrieved were screened by two reviewers, with a sample of 15% screened independently by both reviewers and achieving 98.5% agreement (SH, NJ). Discrepancies were resolved between reviewers. For full text screening, ten reviews (7%) were initially screened by three reviewers (SH, NJ, SW), with results later discussed and discrepancies resolved. Following this, the full text screening was divided across reviewers and any reviews that were not a clear exclude/include (20%) were discussed and agreed between reviewers. This meant that, across all reviews screened by full text, 27% were discussed and agreed by more than one reviewer. Where full texts could not be accessed, authors were contacted to request the full text.

**Fig. 1:**
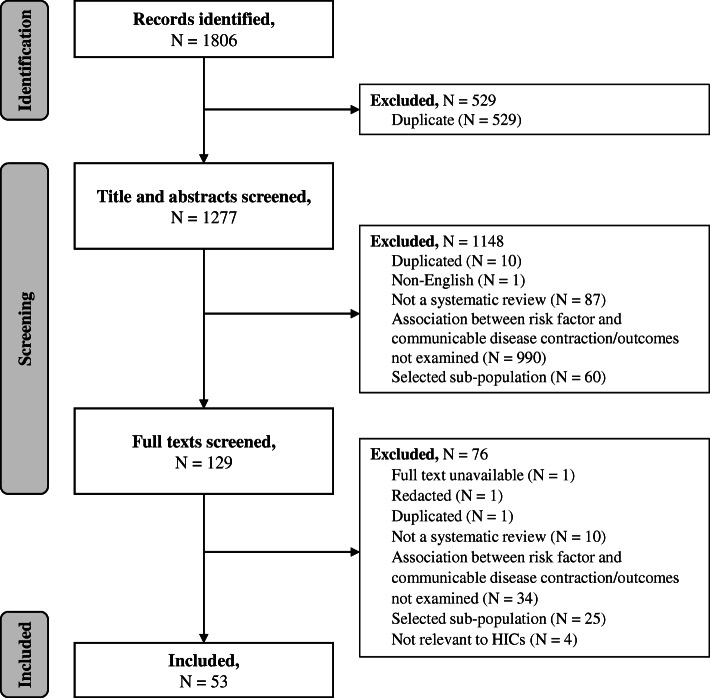
PRISMA flow diagram of study identification, inclusion and exclusion

Systematic reviews and meta-analyses of observational studies (including cohort and case-control studies) that examined the association between an identified behavioural risk factor and the contraction or outcomes of an identified communicable disease (including infection or related pathogens) were included in the review. Since there are no single definitions of the selected risk factors across the literature, all reviews that focused on an identified risk factor were included regardless of the definition used in the review (definitions are provided in Tables 1 and 2 and in the results section). An aim of the review was to explore risks associated with illicit drug use in general. However, it was recognised that injection drug use can be a mechanism of transmission for some pathogens relating to included communicable diseases (e.g. HIV, HCV). Studies that focused specifically on injection drug use were therefore included alongside those focusing on drug use more generally. Studies were excluded if they: were not a systematic review or meta-analysis; did not examine the association between an selected behavioural risk factor and the contraction or outcomes of an selected communicable disease; included only selected specialist sub-populations (e.g. sex workers, prisoners), or included sub-populations relating to a risk factor (e.g. people who inject drugs) without a general population comparison; or included behavioural risk factors or communicable diseases not relevant to HICs (see above). No restrictions were made for the age of participants included. A flow chart demonstrating the selection process is presented in Fig. 1.

**Table 1:** Results and main conclusions relating to identified risk factors from included studies: risk of contraction

Disease contracted	Ref	Definition of risk factor	Meta-analysis findings		No. of studies^a^	Conclusion^b^
			Comparison group	Effect size (95% CI)		Effect direction
**Alcohol as a risk factor**						
TB	[18]	Any alcohol use or higher amounts	No alcohol or lower amounts	OR 1.90 (1.63–2.23)	44	↑
	[19]	Alcohol consumption	N/A		Unclear	–
HIV	[20]	Binge drinking or alcohol misuse	N/A		2	**↑**
Pneumonia	[21]	Any alcohol use or higher amounts	No alcohol or lower amounts	RR 1.83 (1.30–2.57)	14	↑
	[22]	AUD	No AUD	RR 8.22 (4.85–13.95)	2	↑
IBD (IPD)	[23]	Alcohol consumption	N/A		6	↑
**Illicit drug use as a risk factor**						
TB	[19]	Drug abuse	–		Unclear	–
	[24]	Injection drug use	–		Unclear	↑
HIV	[20]	Injecting drugs, smoking crack cocaine	–		7	↑
	[25]	Illicit drug use	–		7	↑
	[24]	Injection drug use	–		Unclear	↑
HCV	[26]	PWID	Community based studies and blood donors	Prevalence of HCV:		↑
				PWID 44.71% (37.5–52.03)	46	
				Community 0.85% (0.00–3.98)	4	
				Blood donors 0.44% (0.40–0.49)	211	
	[27]	PWID	General population	Prevalence of HCV:		↑
				PWID 52.2% (46.9–57.5)	56	
				General pop. 0.3% (0.2–0.4)	122	
	[28]	PWID	General population	Prevalence of HCV:		↑
				PWID 53.6% (36.2–70.6)	15	
				General pop. 6.2% (5.7–6.7)	148	
	[29]	PWID	–		7	↑
**Obesity as a risk factor**						
Influenza	[30]	Obesity and morbid obesity	–		7	↑
Pneumonia	[31]	Obesity (BMI: 30–39.9 kg/m2)	Normal weight	CAP: RR 1.03 (CI 0.8–1.3)	10	↑
				Influenza related: RR 1.31 (1.05–1.63)	10	
				Nosocomial: RR 1.26 (0.80–1.98)	5	
	[32]	Overweight and obesity	Normal weight	RR 1.33 (95% CI 1.04–1.71)	13	↑
**Smoking as a risk factor**						
HIV	[20]	Current smoking	–		1	↑
Pneumonia	[33]	a) Current smoking	Never smokers	a) OR 2.17 (1.70–2.76)	13	↑
		b) Ever smoked		b) OR 2.31 (1.99–2.69)	13	
IBD (IPD)	[23]	Current or former smoking	–		6	↑
**Second-hand smoke as a risk factor**						
TB	[34]	Exposure to ETS	No exposure to ETS	TB infection (children): OR 1.9 (0.9–2.9)	3	↑
				TB disease (children): OR 2.8 (0.9–4.8)	5	
	[35]	Exposure to SHS	No exposure to SHS	TB infection (children): RR 1.19 (0.90–1.57)	6	↑—
				TB disease: RR 1.59 (1.11–2.27)	6	
	[36]	Exposure to SHS	No exposure to SHS	TB infection: RR 1.67 (1.12–2.48) and adjusting for age/SES, RR 1.11 (0.90–1.57)	6	–
				TB disease: RR 1.96 (1.37–2.80) and adjusting for age/SES, RR 2.13 (1.18–3.83)	12	
Pneumonia	[33]	Exposure to ECS	No exposure to ECS	Aged 65+: OR 1.64 (1.17–2.30)	2	↑—
				All ages: OR 1.13 (0.94–1.36)	5	
IBD	[37]	Exposure to SHS	No exposure to SHS	IMD: OR 2.02 (1.52–2.69)	16	↑—
				IHD: OR 1.22 (0.93–1.62) and	12	
				IHD (pre-schoolers) OR 1.46 (1.19–1.81)	9	
				IPD: OR 1.21 (0.69–2.14)	4	
**Physical inactivity as a risk factor**						
Influenza Pneumonia	[38]	Lack of prolonged, moderate aerobic exercise	–		7	↑

^a^ Number of studies for either the meta-analysis or (for narrative reviews) the risk factor. ^b^ Conclusion statements are included in Supplementary file 2. Effect direction is based on conclusions: ↑ = increased risk; ↓ = decreased risk; — = no association. *OR* Odds ratio, *RR* Relative risk, *TB* Tuberculosis, *HIV* Human immunodeficiency virus, *HCV* Hepatitis C virus, *IBD* Invasive bacterial disease, *IPD* Invasive pneumococcal disease, *IMD* Invasive meningococcal disease, *IHD* Invasive Hib disease, *CAP* Community acquired pneumonia, *AUD* Alcohol use disorder, *BMI* Body mass index, *SHS* Second-hand smoke, *ETS* Environmental tobacco smoke, *ECS* Environmental cigarette smoke, *PWID* People who inject drugs

**Table 2:** Results and main conclusions relating to identified risk factors from included studies: risk of more severe outcomes

Disease	Ref	Definition of more severe outcomes	Definition of risk factor (where recorded)	Meta-analysis (MA) findings		No. of studies^a^	Conclusion^b^
				Comparison group	Effect size (95% CI)		Effect direction
**Alcohol as a risk factor**							
TB	[39]	Death	Alcoholism	–		Unclear	↑
	[40]	Treatment default, failure or death	Alcohol misuse	No alcohol misuse	RR 1.45 (1.21–1.74)	15	↑
	[41]	MDR TB	Alcohol use	–		8	↑
	[19]	Poor outcomes	Alcohol use	–		Unclear	–
	[42]	Relapse after treatment	Alcoholism	–		1	↑
	[43]	Unsuccessful treatment	Alcohol use	No alcohol use	OR 2.0 (1.67–2.50)	9	↑
	[44]	Death, treatment failure, lost to follow up	Alcohol use	No or low alcohol use	DS-TB OR 1.99 (1.57–2.51)	25	↑
					MDR-TB OR 2.00 (1.73–2.32)	18	
HIV	[45]	Poor treatment outcomes	Alcohol use disorders	–		10	↑
	[46]	Viral non-suppression	Alcohol use	No alcohol use	OR 2.47 (1.58–3.87)	6	↑
	[47]	HIV progression, survival	Alcohol use	–		17	~
HCV	[48]	No spontaneous clearance	Current/history of excess use	No history of excess use	OR 1.49 (1.05–2.13)	5	↑
	[49]	No achievement of SVR	Alcohol use	–		10	↑
**Illicit drug use a risk factor**							
TB	[39]	Death	Injection drug use	–		Unclear	↑
	[41]	MDR-TB	Drug abuse	–		7	↑
	[19]	Poor outcomes	Drug abuse	–		Unclear	–
HIV	[50]	AIDS-related mortality	Regular/problem cocaine use	General pop.	SMR 23.12 (11.30–47.31)	6	↑
HCV	[48]	No spontaneous clearance	History of injection drug use	Non-injecting drug use	OR 1.69 (1.08–2.70)	7	↑
	[51]	Reinfection with HCV	Recent drug use	Receiving OAT with no recent drug use	OAT: aRR 3.50 (1.62–7.53)	21	↑
					No OAT: aRR 3.96 (1.82–8.59)	15	
	[52]	No achievement of SVR	Recent People who inject drugs (PWID)	Non recent PWID	RR 1.01 (0.95–1.08)	10	–
HBV/HDV	[53]	Infection with HDV (among those with HBV)	Intravenous drug users (IVDUs)	Mixed population, no risk factors	HDV seroprevalence:		↑
					IVDUs 37.57% (29.30–46.20)	44	
					Mixed pop. 10.58% (9.14–12.11)	177	
**Obesity as a risk factor**							
Influenza	[54]	ICU admission or death	a) Obesity, BMI ≥30 kg/m^2^	a) Not obese	a) OR 2.14 (0.92–4.99)	5	↑
			b) Morbid obesity ≥40 kg/m^2^	b) Not morbidly obese	b) OR 2.01 (1.29–3.14)	5	
	[30]	Higher level of healthcare	Obesity and morbid obesity	–		7	↑
	[55]	Death	Obesity BMI > 30 kg/m2	Not obese	OR 2.74 (1.56–4.80)	33	↑
	[56]	a) Hospital admission	Obesity BMI > 30 kg/m2	Not obese	a) RR 1.82 (1.48–2.24)	15	↑
		b) ICU admission or death			b) Adults: RR 1.40 (1.01–1.95),	24	
					Children: RR 0.91 (0.47–1.74)	8	
COVID-19	[57]	Poor outcomes	Obesity			9	↑—
	[58]	ICU admission, IMV or death	Obesity	Non-obese	RR 1.40 (0.91–2.17)	3	↑
	[59]	Worse outcomes	Obesity or overweight			20	↑
	[60]	a) ICU admission	Obesity (BMI Asians > 25 kg	Non-obese	a) OR 1.21 (1.00–1.46)	6	↑
		b) IMV	/m^2^, Caucasians > 30 kg/m^2^)		b) OR 2.05 (1.16–3.64)	5	
Pneumonia	[32]	Mortality	Overweight and obesity	Normal weight	RR 0.83 (0.77–0.91)	10	↓
**Smoking as a risk factor**							
TB	[39]	Death	Smoking			Unclear	–
	[41]	DR TB	Smoking			13	↑
	[19]	Poor outcomes	Smoking			Unclear	↑
	[43]	Unsuccessful treatment	Smoking			16	↑
	[40]	Treatment default, failure or death	Smoking	Non-smokers	RR 0.94 (0.75–1.19)	11	–
	[61]	a) Unfavourable outcomes	Current cigarette smoking	Non-smokers	a) OR 1.23 (1.14–1.33)	8	↑
		b) Delayed smear/culture conversion			b) OR 1.55 (1.04–2.07)	5	
	[42]	Relapse after treatment	Smoking			2	↑
	[62]	DR TB	Current/past smoking	Non-smokers	OR 1.57 (1.33–1.86)	33	↑
Influenza	[56]	a) Hospital admission	Tobacco smoking	Non-tobacco smokers	a) RR 1.24 (1.07–1.43)	10	↑
		b) ICU admission or death			b) RR 1.46 (1.25–1.69)	30	
COVID-19	[63]	Adverse outcomes	Current smoking	Non-current smokers	OR 1.53 (1.06–2.20)	18	↑
	[64]	ARDS, ICU admission or death	Smoking	Non-smokers	OR 1.54 (1.07–2.22)	5	↑
	[65]	ICU, severe oxygenation, IMV, death	Current smoking	Past/never smokers	RR 1.45 (1.03–2.04)	2	↑
	[66]	ICU admission or death	a) History of smoking	Non-smokers	a) OR 2.17 (1.37–3.46)	16	↑
			b) Current smoking		b) OR 1.51 (1.12–2.05)	10	
	[67]	ICU, IMV or death	Active smoking	Non-smokers	All studies OR 1.98 (1.29–3.05)	7	↑—
					One removed OR 1.55 (0.83–2.87)	6	
	[68]	Worsening of symptoms, ICU, death	a) Current smoking	Non-smokers	a) OR 1.98 (1.16–3.39)	13	↑
			b) Former smoking		b) OR 3.46 (2.46–4.85)	4	
**Second-hand smoke as a risk factor**							
Pneumonia	[69]	Death from ALRIs (incl. pneumonia)	SHS exposure	No SHS exposure	(Children) OR 1.52 (1.20–1.93)	8	↑
**Poor diet as a risk factor**							
HIV	[70]	HIV progression, mortality	Vit.D deficiency/insufficiency			10	↑
HCV	[49]	No achievement of SVR	Poor quality diet			2	–

^a^ Number of studies for either the meta-analysis or (for narrative reviews) the risk factor. ^b^ Conclusion statements are included in Supplementary file 2. Effect direction is based on conclusions: ↑ = increased risk; ↓ = decreased risk; — = no association; ~ = variable results. *OR* Odds ratio, *RR* Relative risk, *aRR* Adjusted rate ratio, *SMR* Standardised mortality ratio, *TB* Tuberculosis, *DR-TB* Drug-resistant tuberculosis, *MDR-TB* Multi-drug resistant tuberculosis, *DS-TB* Drug-susceptible tuberculosis, *HIV* Human immunodeficiency virus, *AIDS* Acquired immunodeficiency syndrome, *HCV* Hepatitis C virus, *HBV* Hepatitis B virus, *HDV* Hepatitis D virus, *ARDS* Acute respiratory distress syndrome, *ARLI* Acute lower respiratory infection, *PWID* People who inject drugs, *IVDU* Intravenous drug user, *SHS* Second-hand smoke, *BMI* Body mass index, *SVR* Sustained virological response, *ICU* Intensive care unit, *IMV* Invasive mechanical ventilation, *OAT* Opioid agonist therapy

### Data extraction and synthesis

Data were extracted by three reviewers (SH, NJ, SW) into a standardised, pre-piloted form. Each extraction was duplicated across reviewers and discrepancies resolved through discussion. Information extracted from the studies included: title, authors, abstract, behavioural risk factor(s) studied, communicable disease(s) studied (including infection or related pathogens), research question, geographical restrictions, population characteristics, number of reviews included in the systematic review or meta-analysis, main findings (including odds ratios (OR), relative risks (RR) or rate ratios where available), proposed mechanisms of association and conclusions related to identified behavioural risk factor(s) and communicable disease(s). Where information on the number of reviews included for each risk factor was not reported, the corresponding author of the paper was contacted for additional information.

Due to the variety of different communicable diseases, risk factor definitions, outcome measures, and methods of reporting in the included studies, as well as the challenges of conducting meta-analysis for observational studies [71], findings were not combined statistically through meta-analysis. Instead, a narrative synthesis of the findings was constructed [72], and effect size ranges reported for each behavioural risk factor. Key information that would have enabled calculation of a common effect size was often not available. To calculate these effect size ranges, it was assumed that ORs, RRs and rate ratios were approximately equivalent, a method suggested for umbrella reviews in these circumstances [73]. In addition, where studies reported reduced risk of a communicable disease with a health behaviour (e.g. physical activity, no alcohol drinking), an inverse OR (1/OR) for the corresponding risk behaviour was reported. Findings were structured according to the identified behavioural risk factors and their association with a) contraction of the identified communicable diseases, and b) experiencing more severe outcomes from these communicable diseases. Some study conclusions were amended for readability, to aid understanding. Further, where study conclusions were not relevant to the current research question, information was extracted from results sections and amended for readability (see Supplementary file 2).

### Methodological quality of studies

The methodological quality of included studies was assessed using the Overview Quality Assessment Questionnaire (OQAQ); a frequently used, validated tool for assessing the methodological quality of systematic reviews [74]. Methodological quality assessment was carried out by three researchers (NJ, SH, SW), with any discrepancies resolved through discussion. Assessment ratings are available in Supplementary file 2.

## Results

The database search yielded 1806 citations, of which 53 were included (Fig. 1). Research relating to the following communicable diseases (including infection and pathogens) was identified: TB, HIV, hepatitis C virus (HCV), hepatitis B virus (HBV), invasive bacterial disease (IBD), pneumonia, influenza, and COVID-19. No studies relating to SARS, MERS or meningitis were identified. Thirty-six of the identified systematic reviews also conducted meta-analyses. Reviews used a range of definitions of behavioural risk factors (e.g. current or former smoker, any alcohol use or heavy alcohol use). All definitions were included in the synthesis, and are presented for clarity in each section of the results and in the results tables (Tables 1 and 2). Eighteen reviews examined the association between behavioural risk factors and the contraction of a communicable disease only, 32 reviews examined the association between behavioural risk factors and the outcomes from communicable diseases only, and three reviews examined associations with both contraction of and outcomes from communicable diseases. Characteristics of all included reviews and their conclusions can be found in Supplementary file 2. No systematic review had extensive or major flaws, with most reviews having only minimal or minor flaws (Supplementary file 2). Consequently, no reviews were excluded based on methodological quality. A breakdown of reviews by disease and risk factors is provided in Supplementary file 3.

### Behavioural risk factors for communicable diseases

**Fig. 2:**
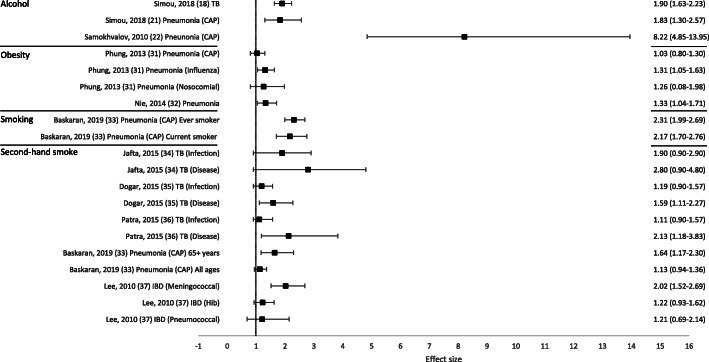
Forest plot of meta-analysis effect sizes: contraction of a communicable disease. Effect sizes refer to odds ratios and relative risks, see Table 1 for more information. CAP = community acquired pneumonia; TB = tuberculosis; IBD = invasive bacterial disease

**Fig. 3:**
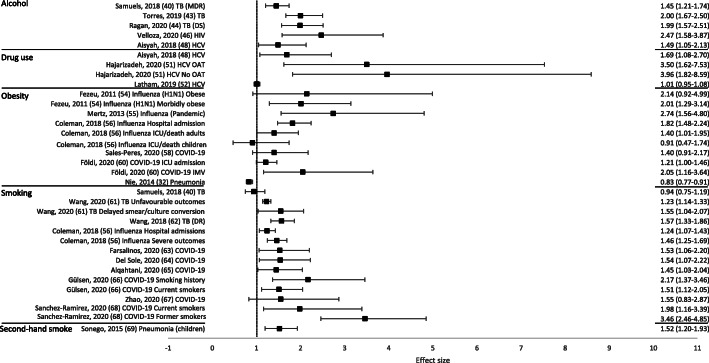
Forest plot of meta-analysis effect sizes: more severe communicable disease outcomes. Effect sizes refer to odds ratios, relative risks and rate ratios, see Table 2 for more information. MDR = multi-drug resistant; TB = tuberculosis; DS = drug-susceptible; HIV = human immunodeficiency virus; HCV = hepatitis C virus; ICU = intensive care unit; IMV = invasive mechanical ventilation; OAT = opioid agonist therapy

Overall, 50 out of 53 reviews (94%) concluded that at least one of the behavioural risk factors studied increased the risk of contracting or having more severe outcomes of a communicable disease. Across all reviews, effect sizes, where calculated, ranged from 0.83 to 8.22 (Figs. 2 and 3; Tables 1 and 2). Nineteen out of 21 reviews (90%) concluded that at least one of the behavioural risk factors studied increased the risk of contracting a communicable disease (Table 1). Across all contraction reviews, effect sizes, where calculated, ranged from 1.03 to 8.22 (Fig. 2). Thirty-two out of 35 reviews (91%) concluded that at least one of the behavioural risk factors studied increased the likelihood of having more severe outcomes from a communicable disease (Table 2). Across all outcome reviews, effect sizes, where calculated, ranged from 0.83 to 3.96 (Fig. 3).

### Alcohol as a risk factor

Seventeen reviews included alcohol as a risk factor for a communicable disease, with a range of definitions used: any alcohol consumption [19, 23, 41, 43, 44, 46, 47, 49]; any alcohol use or higher amounts [18, 21]; binge drinking or alcohol misuse [20]; alcohol misuse [40]; alcohol use disorder (AUD) [22, 45]; alcoholism [39, 42]; or current/history of excess use [48]. The majority of reviews reported an increased risk of contraction (5/6 reviews; Table 1) and more severe outcomes (10/12 reviews; Table 2). Across all alcohol reviews, effect sizes, where calculated, ranged from 1.83–8.22 for contraction (Fig. 2) and 1.45–2.47 for severe outcomes (Fig. 3). Alcohol use (any use, higher amounts, binge drinking or AUD) was reported to increase the risk of contracting TB [18], HIV [20], pneumonia [21, 22] and invasive pneumococcal diseases (IPD) [23]. One review did not draw a conclusion, but reported mixed findings for the association between alcohol consumption and contraction of TB [19]. Alcohol use (any use, misuse, current/history of excess use, alcoholism or AUD) was reported to increase the risk of having more severe outcomes from TB [39–44], HIV [45, 46], and HCV [48, 49]. One review reported mixed findings and made no clear conclusion about the association of alcohol consumption and TB outcomes [19], and one review reported variable results among studies examining the association between alcohol consumption and the progression of HIV [47].

### Illicit drug use as a risk factor

Fifteen reviews examined the association between illicit drug use and communicable disease contraction or outcomes, with a wide range of definitions used: drug abuse [19, 41]; illicit drug use [25]; regular/problem cocaine use [50]; recent drug use [51]; and injection drug use [20, 24, 26–29, 39, 48, 52, 53]. The majority of reviews reported an increased risk of contraction (8/9 reviews; Table 1) and more severe outcomes (6/8 reviews; Table 2). Across all drug use reviews, effect sizes, where calculated (for more severe outcomes only), ranged from 1.01–3.96 (Fig. 3). Both injection drug use and illicit drug use were reported to increase the risk of contracting TB [24] and HIV [20, 24, 25], whilst the prevalence of HCV was found to be higher among people who inject drugs (PWID) compared to general population or community groups [26–29]. One review did not draw a conclusion but reported mixed findings for the association between drug abuse and TB contraction [19]. Both injecting drug use and drug use/abuse were reported to increase the risk of having more severe outcomes from TB [39, 41], HIV [50] and HCV [48, 51]. Furthermore, among those with HBV, the prevalence of hepatitis D (HDV; co-infection with HDV is considered a more severe form of viral hepatitis) was substantially higher for PWID compared to a mixed population with no risk factors [53]. One review did not draw a conclusion but reported mixed findings for the association between drug abuse and TB outcomes [19], and one review concluded that treatment outcomes for HCV were similar between people who currently were and were not injecting drugs [52].

### Physical inactivity as a risk factor

One systematic review was identified examining the association between physical activity and communicable disease contraction or outcomes. This study reported an association between increased prolonged, moderate aerobic exercise and reduced influenza-related mortality, and improved immunocompetence [38].

### Obesity as a risk factor

Ten reviews focused on the relationship between obesity [30, 31, 54–58, 60], or overweight and obesity [32, 59], and communicable disease risk. The majority of reviews reported an increased risk of contraction (3/3 reviews; Table 1) and more severe outcomes (8/9 reviews; Table 2). Across all obesity reviews, effect sizes, where calculated, ranged from 1.03–1.33 for contraction (Fig. 2) and 0.83–2.74 for severe outcomes (Fig. 3). Obesity was reported to increase the risk of contracting influenza [30] and pneumonia [31, 32]. Obesity was reported to increase the risk of having more severe outcomes from influenza [30, 54–56] and COVID-19 [58–60]. One review concluded that most studies showed some degree of association between higher body mass index (BMI) and a worse clinical presentation of COVID-19 and the need for hospitalisation. This review suggested that obesity seemed to predict poor clinical evolution in patients with COVID-19, but that studies in the review had limited methodological quality [57]. However, one review, which concluded that obesity increased the risk of contracting pneumonia, also found that obese individuals had a lower mortality risk from pneumonia [32].

### Smoking as a risk factor

Eighteen reviews examined the association between smoking (current, past or both) and communicable disease contraction or outcomes. The majority of reviews reported some evidence of an increased risk of contraction (3/3 reviews; Table 1) and more severe outcomes (13/15 reviews; Table 2). Across all smoking reviews, effect sizes, where calculated, ranged from 2.17–2.31 for contraction (Fig. 2) and 0.94–3.46 for severe outcomes (Fig. 3). Smoking was reported to increase the risk of contracting HIV [20], pneumonia [33] and invasive pneumococcal disease (IPD) [23]. Further, smoking was reported to increase the risk of having more severe outcomes from TB [19, 41–43, 61, 62], influenza [56], and COVID-19 [63–66, 68]. Two reviews reported no associations between smoking and more severe outcomes from communicable diseases, including death from TB [39] and TB treatment outcomes [40]. One review reported that active smoking may increase the risk of severe COVID-19, but found the result was heavily influenced by one study [67].

### Second-hand smoke as a risk factor

Six reviews focused on second-hand smoke as a risk factor for a communicable disease. The majority of reviews reported some evidence of an increased risk of contraction (4/5 reviews; Table 1) and more severe outcomes (1/1 review; Table 2). Across all second-hand smoking reviews, effect sizes, where calculated, ranged from 1.11 to 2.80 for contraction (Fig. 2), and the one effect size calculated for severe outcomes was 1.52 (Fig. 3). One review suggested that second-hand smoke exposure increased the risk of TB infection and disease [34]. The remaining four reviews reported at least some evidence of second-hand smoke exposure increasing the risk of contracting a communicable disease, including TB [35, 36], pneumonia (among those aged 65+ only) [33], and IBD (invasive meningococcal disease [IMD] only) [37]. Second-hand smoke exposure was reported to increase the risk of severe outcomes from acute lower respiratory infections (ALRIs), including pneumonia [69].

### Poor diet as a risk factor

Only two reviews were identified that examined the association between poor diet and communicable disease outcomes, and no reviews examining the association between poor diet and communicable disease contraction were identified. One review found that vitamin D status may influence the course of HIV disease [70]. The second review reported that a high intake of polyunsaturated fatty acids was associated with non-response to HCV antiviral therapy [49].

## Discussion

The key finding of this systematic review is that behavioural risk factors play a significant role in the risk of contracting, and having more severe outcomes from, common communicable diseases. To the authors’ knowledge, this is the first time that a review has brought together studies exploring the impact of behavioural risk factors on a range of communicable diseases. Whilst the focus on selected communicable diseases and use of systematic reviews has led to inevitable gaps, the findings nevertheless provide strong evidence that both NCDs and communicable diseases share a common set of behavioural risk factors. This work indicates that the prevention of communicable disease is likely to be most successful if it involves the prevention of behavioural risk factors. These findings are timely, in light of the COVID-19 pandemic, and highlight potential additional benefits of addressing behavioural risk factors ahead of any future epidemics or pandemics. While the specific diseases that may be involved can only be speculated, they are likely to share at least some characteristics with diseases in this review.

Although this review has not examined the mechanisms connecting behavioural risk factors and communicable diseases, there are likely to be multiple mechanisms. Behavioural factors, such as alcohol use, smoking, obesity, and illicit drug use, are well documented to impair the immune system. For instance, smoking is known to influence both innate and adaptive immunity [75]. Impairments to the immune system can make individuals more susceptible to communicable diseases and less able to control or recover from infection, leading to worse outcomes [76–81]. Use of alcohol/drugs may also reduce the efficacy of treatment for communicable diseases [82]. The presence of comorbidities, such as diabetes and cardiovascular disease, in individuals with behavioural risk factors has also been implicated in the increased risk of communicable diseases [83, 84]. However, behavioural risk factors, such as obesity, are also reported to independently influence communicable diseases, after adjusting for comorbidities [55]. Behavioural mechanisms may also be important, particularly for alcohol and drug use, which may reduce risk perception [78], interfere with the uptake of services, or lead to poorer treatment adherence [85]. Additionally, behavioural risk factors may be likely to appear in combination, for example combined alcohol use and smoking [86], to further increase influences on communicable diseases. Furthermore, behaviours associated with drug use, such as injecting drugs, have a high efficiency of transmission of communicable diseases and reinfection with communicable diseases [87]. Having a communicable disease could also lead to the presence of behavioural risk factors (e.g. alcohol may be used as a way of coping with the emotional distress of diseases such as HIV and HCV [88]). Finally, there may be social mechanisms, such as the social marginalisation of heavy drinkers that affects health service use or treatment [89], or social issues such as homelessness, incarceration and poverty, which may increase the risk of both behavioural risk factors and communicable diseases [90, 91]. It is likely that there are multiple ways in which these different physiological, behavioural and social factors come together to affect the likelihood of transmission and severity of communicable disease, which require further investigation.

With behavioural risk factors influencing the contraction and severity of communicable diseases, their prevention is likely to play a role in addressing future communicable disease burden, potentially through improvements in the immune system, bodily functioning and risk behaviours. As the recent COVID-19 pandemic has highlighted, their prevention is also likely to impact on communicable disease burden through the potential reduction of NCDs commonly associated with behavioural risk factors, which can also alter immune system function [92] and increase the risk of communicable disease complications and death [10, 93]. The review findings are important in understanding communicable disease risk, and timely, in light of COVID-19. They suggest that improvements in the prevention of behavioural risk factors may serve to reduce the negative impacts of future epidemics or pandemics, building resilience and helping to address the pressing need for greater investment in pandemic preparedness [94]. Indeed, COVID-19 should not only be a reminder that good communicable disease control is necessary, but that the more successful we are in addressing behavioural risk factors, the better we will also be at reducing the burden of communicable disease, including future epidemics or pandemics. The finding that both communicable diseases and NCDs share a common set of behavioural risk factors also lends support for a more holistic understanding of these two disease categories. For instance, research suggests that NCDs and communicable diseases can interact; whilst NCDs can increase the risk and severity of communicable diseases (e.g. individuals with diabetes, hypertension and respiratory illnesses are more likely to affected by COVID-19 [95]), at least some diseases previously considered NCDs are now known to have an infectious origin (e.g. HBV is a cause of heptatocellular carcinoma [96]).

Although the focus of this review is on HICs, findings will be of importance to LMICs, which often experience a much higher burden of communicable disease [9] and where, for many countries, the prevalence of behavioural risk factors is increasing [7, 8]. Due to ageing populations, the negative impacts of globalisation, and ill-equipped health systems, these countries are also facing a rapidly growing burden of NCDs [97–99], which may reduce resistance to infection, increase communicable disease complications, or interfere with its treatment [100, 101]. In the current global society, any negative effects of rising behavioural risk factors and related NCDs on communicable disease transmission have the potential to affect not only LMICs, but health and well-being globally.

Across both HICs and LMICs, behavioural risk factors and related NCDs are known to cluster in disadvantaged populations [97, 102–104], with poverty contributing to behavioural risk factors and NCDs, and vice versa [97]. Disadvantaged communities are more likely, therefore, to experience dual burdens of NCDs and communicable disease, contributing to social and economic health inequalities. In the UK for instance, people living in the most disadvantaged communities have been over twice as likely to die from COVID-19 as those in the least disadvantaged areas [105]. Preventing behavioural risk factors, particularly among disadvantaged populations, is likely to play an important role in reducing future global and national health inequalities, as well as the unequal burden of future pandemics.

There are some limitations to this work. The wide-ranging nature of the research allowed for a broad view of the links between behavioural risk factors and communicable diseases. However, this did not allow for the exploration of causal pathways of specific associations. Further research exploring these pathways would aid understanding and inform prevention. The use of systematic reviews to achieve a broader range of information also meant that newer empirical research may have been missed, only more widely researched topics for which there is enough information to conduct a systematic review would have been included, and more in-depth information such as potential interactions between risk factors could not be included. With no single definitions of behavioural risk factors agreed across the literature, it was not possible to standardise the definitions of risk factors in this review, meaning that there was often variation in the definitions included in each risk factor category, hampering discussion of relationships. Low socio-economic status (SES) and other factors associated with low SES, such as poor housing, are likely to be an important element in the link between behavioural risk factors and communicable diseases, although little is currently known about the influence of low SES and associated factors. It was not possible to explore the role of low SES within the review since many of the reviews included did not explore low SES in their analyses. Many of the included studies are global syntheses, however, the relationships between behavioural risk factors and communicable diseases may vary between countries. Only papers written in English were included, meaning that research in other languages may have been missed. Finally, conclusions should be considered with publication bias in mind; papers are more likely to be published if they reveal significant effects rather than null findings [106], so those reporting that behavioural risk factors are associated with communicable diseases are more likely to be identified.

This work identified several gaps in the current systematic review literature relating to specific behavioural risk factors and common communicable diseases, including studies examining the association of physical inactivity and poor dietary habits with communicable diseases, which warrant urgent further exploration. For instance, recently published literature has indeed highlighted the important role of physical inactivity in severe COVID-19 risk [2, 107]. Due to the study being limited to systematic reviews only, a comprehensive comparison of behavioural risk factors across different disease types could not be provided; although future reviews could provide such comparisons. However, it was noted that reviews examining the association of communicable diseases with alcohol and illicit drug use largely focused on TB, HIV and hepatitis, whereas reviews examining association with obesity largely focused on pneumonia, influenza and COVID-19 (see Supplementary file 3). Further research understanding the more intricate ways in which individual behavioural risk factors are linked to specific types of disease, and the mechanisms by which they are linked, would provide a valuable framework for understanding how current and future communicable diseases may affect different population groups. Finally, findings highlight an opportunity for future research to examine the efficacy of behavioural risk factor prevention efforts in reducing communicable disease burden.

## Conclusions

Behavioural risk factors play a significant role in the risk of contracting, and having more severe outcomes from, common communicable diseases. These risk factors are largely modifiable or preventable. Prevention of communicable diseases is likely to be most successful if it involves the prevention of behavioural risk factors that are commonly associated with NCDs, particularly among disadvantaged populations. These findings are important for understanding risks associated with communicable disease, and timely, given the current COVID-19 pandemic and need for improvements in future pandemic preparedness. Addressing behavioural risk factors should be an important part of work to build resilience against any emerging and future epidemics and pandemics. Furthermore, the pandemic can offer a timely, teachable moment for the public on how improvements to general health, through addressing risk behaviours commonly associated with NCDs, may help protect them from infections like COVID-19 in the future.

## Supplementary Information

**Additional file 1.**

**Additional file 2.**

**Additional file 3.**

## Data Availability

Not applicable.

## Abbreviations

AIDS: Acquired immune deficiency syndrome
ALRI: Acute lower respiratory infections
BMI: Body mass index
HCV: Hepatitis C virus
HDV: Hepatitis D virus
HIC: High-income country
HIV: Human immunodeficiency virus
IBD: Invasive bacterial disease
IPD: Invasive pneumococcal disease
IMD: Invasive meningococcal disease
LMIC: Lower-middle income country
MERS: Middle east respiratory syndrome
NCD: Non-communicable disease
OR: Odds ratio
OQAQ: Overview quality assessment questionnaire
PRISMA: Preferred reporting items for systematic reviews and meta-analyses
PROSPERO: International prospective register of systematic reviews
RR: Relative risk
SARS: Severe acute respiratory syndrome
TB: Tuberculosis
