# Supplementary material for: The impact of behavioural risk factors on communicable diseases: a systematic review of reviews
Source: BMC Public Health. 2021 Nov 17;21:2110. doi: 10.1186/s12889-021-12148-y (PMC8596356; doi:10.1186/s12889-021-12148-y)
Supplement: Supplementary file 1 — Additional file 1. [file 12889_2021_12148_MOESM1_ESM.docx]

**Supplementary File 1: Search strategy**

We searched the following electronic databases through ProQuest: APA PsycInfo, Art, Design & Architecture Collection, British Nursing Database, Coronavirus Research Database, SciTech Premium Collection, and Social Science Premium Collection.

We searched for the following terms in title or abstract: “systematic* review” OR “meta analysis” AND alcohol OR smoker OR smoking OR tobacco OR drug* OR “substance use” OR “substance misuse” OR “physical activity” OR exercise OR “physical inactivity” OR sedentary OR nutrition OR diet OR overweight OR obes* OR “low fruit” OR “low vegetable” OR “hous* condition*” OR “hous* quality” OR “poor heat*” OR “indoor air pollution” OR “indoor air quality” OR (damp AND hous*) AND “communicable disease*” OR “infectious disease*” OR tuberculosis OR HIV OR “human immunodeficiency virus” OR “acquired immunodeficiency syndrome” OR “influenza” OR H1N1 OR SARS OR “SARS-COV-2” OR “COVID 19” OR coronavirus OR MERS OR “MERS-CoV” OR pneumococcal OR pneumonia OR hepatitis OR meningitis AND risk* OR “protective factor*” OR associat* OR link OR relation* OR precursor* OR predispos* OR interact* OR connect* OR influence* OR mediat* OR moderat

We limited searches to peer reviewed articles only, English language and last 10 years (covering the period 28^th^ October 2010 to 28^th^ October 2020).
