# Supplementary material for: The impact of behavioural risk factors on communicable diseases: a systematic review of reviews
Source: BMC Public Health. 2021 Nov 17;21:2110. doi: 10.1186/s12889-021-12148-y (PMC8596356; doi:10.1186/s12889-021-12148-y)
Supplement: Supplementary file 2 — Additional file 2. [file 12889_2021_12148_MOESM2_ESM.docx]

**Supplementary File 2: Study characteristics, conclusions and quality assessment**

The following table presents study characteristics, conclusions and quality assessment for all papers included in the review. The Overview Quality Assessment Questionnaire (OQAQ) was used to quality assess systematic reviews and meta-analyses included in the review. The OQAQ has 9 questions (see A-I below), with each assessment question rated with, “yes (Y)”, “partially (P)”, “can’t tell (CT)” or “no (N)”. There is also an overall assessment score (J) of the scientific quality of the paper, where one equates to “extensive flaws” and seven equates to “minimal flaws”.

1. Were search methods reported?
2. Was the search comprehensive?
3. Were the inclusion criteria reported?
4. Was selection bias avoided?
5. Were the validity criteria reported?
6. Was validity assessed appropriately?
7. Were methods used to combine studies reported?
8. Were findings combined appropriately?
9. Were conclusions supported by the reported data?
10. What was the overall scientific quality of the overview?*

**Study characteristics, conclusions and quality assessment for all included systematic reviews and meta-analyses**

|  | **Study characteristics** | | | | | **Conclusion** | | **Quality assessment** | | | | | | | | | |
| --- | --- | --- | --- | --- | --- | --- | --- | --- | --- | --- | --- | --- | --- | --- | --- | --- | --- |
| **Author, year** | **MA** | **Country** | **Disease** | **Population (*any age restriction*)** | **Main conclusions relating to risk factors and overall effect direction*** | | | **A** | **B** | **C** | **D** | **E** | **F** | **G** | **H** | **I** | **J** |
| Aisyah et al., 2017 (39) | ✓ | Global | HCV | Patients with untreated HCV *(adults)* | **Injecting drug users** or those with **excess alcohol use** were more likely to not spontaneously clear HCV. | | ↑ | Y | Y | Y | Y | Y | Y | Y | Y | Y | 7 |
| Al Kanaani et al., 2018 (46) | ✓ | Pakistan | HCV | PWID and general population | Results indicated a high HCV prevalence among **PWID**. | | ↑ | Y | Y | Y | CT | Y | Y | Y | Y | Y | 6 |
| Alqahtani et al., 2020 (66) | ✓ | China, US | COVID-19 | Patients with confirmed COVID-19 *(child studies excluded)* | **Current smokers** had greater risk of severe complications and a higher COVID-19 mortality rate, compared to former/never smokers. | | ↑ | Y | Y | P | Y | Y | Y | Y | Y | Y | 7 |
| Azar et al., 2010 (36) |  | France, US | HIV | HIV infected individuals | Findings consistently supported an association between **alcohol use disorders** and poor HIV treatment outcomes. | | ↑ | Y | Y | Y | CT | N | N | N/A | Y | Y | 5 |
| Azhar, 2012 (38) |  | India | TB | Patients with successful TB treatment. | **Smoking** and **alcoholism** were risk factors for TB relapse after successful treatment. | | ↑ | Y | N | Y | CT | Y | Y | N/A | Y | Y | 5 |
| Baskaran et al., 2019 (61) | ✓ | Global | Pneumonia (CAP) | Patients with confirmed CAP *(*≥ *15 years old)* | **Current and ex-smokers** were at greater risk of developing CAP, whilst **passive tobacco smoke exposure** had a significant effect only in those aged > 65 years. | | ↑- | Y | Y | Y | Y | Y | Y | Y | Y | Y | 7 |
| Chen et al., 2019 (49) | ✓ | Global | HDV | HBsAG-positive population *(child studies excluded)* | HDV prevalence was substantially higher in an **intravenous drug use** population than in a mixed population. | | ↑ | Y | Y | Y | CT | N | N | Y | Y | Y | 5 |
| Coleman et al., 2018 (54) | ✓ | Global | Influenza | Patients with influenza | **Obesity** was an important predictor of hospital admission and severe outcomes from Influenza. Furthermore, smoking increased the risk of hospital admission and severe outcomes. | | ↑ | Y | Y | Y | Y | Y | Y | Y | Y | Y | 7 |
| Cruickshank et al., 2014 (24) |  | Australia, Canada, US | IBD (IPD) | Various, including hospital and community populations | **Alcohol misuse** and **smoking** may increase the risk of IPD in adults. | | ↑ | Y | Y | Y | Y | Y | Y | N/A | Y | Y | 7 |
| de Siqueira et al., 2020 (60) |  | Global | COVID-19 | Patients with COVID-19 *(adults)* | Being **obese** or overweight was associated with worse outcomes from COVID-19. | | ↑ | Y | Y | Y | CT | N | CT | N/A | Y | Y | 5 |
| Del Sole et al., 2020 (65) | ✓ | China, the Netherlands | COVID-19 | Patients with COVID-19 | **Smoking** was associated with severity. | | ↑ | Y | Y | Y | Y | N | N | Y | Y | Y | 5 |
| Dogar et al., 2015 (71) | ✓ | Global | Pulmonary TB infection and disease | People with no history of TB, non-smokers | There was moderate evidence of an association between **SHS** and risk of developing TB disease, and limited evidence for an association between **SHS** and risk of TB infection. | | ↑- | Y | Y | Y | Y | Y | Y | Y | Y | Y | 7 |
| Fadlalla et al., 2015 (47) |  | The Maghreb (NW Africa) | HCV | PWID and general/hospital populations | **Injection drug use** appeared to be a major, though not dominant, contributor to HCV transmission. | | ↑ | Y | Y | Y | Y | N | N | Y | Y | Y | 5 |
| Falagas et al., 2010 (52) |  | Southern Hemisphere | Pandemic Influenza A (H1N1) | Patients with pandemic A (H1N1) influenza | A substantial proportion of the cases of influenza reported in the studies were in **obese** individuals. Obesity and morbid obesity were more commonly reported as the level of healthcare increased. | | ↑ | Y | Y | Y | Y | N | N | N/A | Y | Y | 5 |
| Farsalinos et al., 2020 (64) | ✓ | Global (mostly China) | COVID-19 | Hospitalised COVID-19 patients | **Current smokers** had higher odds of an adverse COVID-19 outcome compared with non-current smokers, but lower odds compared with former smokers. | | ↑ | Y | Y | Y | CT | Y | Y | Y | Y | Y | 6 |
| Fezeu et al., 2011 (51) | ✓ | Global | Pandemic influenza A (H1N1) | Hospitalised patients with pandemic influenza A (H1N1) | **Obesity** was associated with higher risks of ICU admission or death in patients with influenza A (H1N1) infection. | | ↑ | Y | Y | Y | CT | Y | Y | Y | Y | Y | 6 |
| Földi et al., 2020 (57) | ✓ | Global | COVID-19 | COVID-19 patients with no conditions that influence weight | **Obesity** was a risk factor for ICU admission and IMV requirement. | | ↑ | Y | Y | Y | Y | Y | Y | Y | Y | Y | 7 |
| Ge et al., 2018 (29) |  | Global | HIV | HIV infected individuals *(average age 40+)* | Associations between **alcohol use** and response to treatment (progression of HIV) were variable. | | - | Y | Y | Y | N | Y | Y | N/A | Y | Y | 6 |
| Giusti et al., 2011 (75) |  | Global | HIV | HIV infected patients | Two well-designed trials support that the course of HIV may be influenced by **vitamin D status**. | | ↑ | Y | Y | Y | N | N | N | N/A | Y | Y | 5 |
| Goel et al., 2018 (44) | ✓ | India | HCV | General and selected populations | Anti-HCV prevalence was higher among **PWID.** | | ↑ | Y | Y | Y | CT | N | N | Y | Y | Y | 5 |
| Gülsen et al., 2020 (67) | ✓ | China, USA | COVID-19 | Patients with COVID-19 *(adults)* | **Active smoking** and **history of smoking** were associated with increased severity. | | ↑ | Y | Y | Y | Y | Y | Y | Y | Y | Y | 7 |
| Hajarizadeh et al., 2019 (42) | ✓ | Global | HCV | Patients with successful HCV treatment | HCV reinfection risk following treatment was higher amongst people with **recent drug use** and lower amongst those receiving opioid agonist therapy with no recent drug use. | | ↑ | Y | Y | Y | Y | Y | Y | Y | Y | Y | 7 |
| Jafta et al., 2015 (70) | ✓ | Global | TB infection and disease | Children with TB and those at risk of acquiring it *(children)* | Exposure to **environmental tobacco smoke** increased the risk of childhood TB disease and TB infection. | | ↑ | Y | Y | Y | CT | Y | Y | Y | Y | Y | 6 |
| Latham et al., 2019 (48) | ✓ | Global | HCV | Patients treated for chronic HCV | Treatment outcomes were similar in **recent PWID** compared to non-PWID treated with direct-acting antivirals. | | - | Y | Y | Y | Y | Y | Y | Y | Y | Y | 7 |
| Lee et al., 2010 (73) | ✓ | Global | IBD (IMD, IPD, IHD). | General and hospital populations *(children)* | **SHS** exposure may be associated with IMD. Evidence was insufficient to show an association between SHS and IHD/IPD. | | ↑- | Y | Y | Y | Y | Y | CT | Y | Y | Y | 6 |
| Mahmud et al., 2018 (45) | ✓ | Iran | HCV | General population | HCV prevalence was higher in **PWID** compared to the general population. | | ↑ | Y | Y | Y | CT | Y | Y | Y | Y | Y | 6 |
| Mertz et al., 2013 (53) | ✓ | Global | Influenza | Individuals with influenza infection | **Obesity** was an important cause of death with pandemic and seasonal influenza. However, the level of evidence was low. | | ↑ | Y | Y | Y | Y | Y | Y | Y | Y | Y | 7 |
| Mohidem et al., 2018 (23) |  | Malaysia | TB infection and disease | Not given | No clear conclusions drawn for **alcohol** or **drug use**. Authors suggested that results support increased susceptibility of **smokers** to poor treatment outcomes and prognosis with TB. | | ↑- | Y | Y | Y | CT | N | N | N/A | Y | P | 4 |
| Nie et al., 2014 (59) | ✓ | Global | Pneumonia (Hospital acquired and CAP) | Not given | An ‘obesity survival paradox’ exists for pneumonia: **obese** individuals might have a higher risk of contracting pneumonia but a lower mortality risk. | | ↑↓ | Y | Y | Y | Y | Y | Y | Y | Y | Y | 7 |
| Patra et al., 2015 (72) | ✓ | Global | TB infection and disease | General and hospital populations | **SHS** **exposure** increased the risk of LTBI and active TB after controlling for age, biomass fuel use and contact with a TB patient. There was no significant association of SHS with LTBI after adjustment for SES and study quality. Analysis may not show sufficient evidence to confirm an association. | | - | Y | Y | Y | Y | Y | Y | Y | Y | Y | 7 |
| Peacock et al., 2020 (41) | ✓ | Global | HIV | People with regular or problematic cocaine use in various settings | There were elevated rates of mortality among **people with regular or problematic cocaine use** for deaths attributable to infectious disease (including AIDS). | | ↑ | Y | Y | Y | Y | Y | Y | Y | Y | Y | 7 |
| Peres et al., 2020 (55) |  | Global | COVID-19 | Patients with confirmed COVID-19 | **Obesity** is likely to be a predictor of poor COVID-19 outcomes. | | ↑- | Y | Y | Y | Y | Y | Y | Y | Y | Y | 7 |
| Phung et al., 2013 (58) | ✓ | Global | Pneumonia (CAP, influenza related or nosocomial) | Not given *(≥ 12 years)* | **Obese** persons had a non-significant increased risk of CAP. Obese and morbidly obese persons had an increased risk of influenza-related pneumonia. | | ↑ | Y | Y | Y | CT | Y | Y | Y | Y | Y | 6 |
| Pimpin et al., 2011 (43) |  | EU/EEA | HIV-TB co-infection | Patients with TB | **Injecting drug users** were a high-risk group for TB-HIV co-infection. | | ↑ | Y | Y | Y | Y | N | N | N/A | Y | Y | 5 |
| Ragan et al., 2020 (27) | ✓ | Global | TB (DS or MDR-TB) | Patients treated for DS or MDR-TB disease *(child studies excluded)* | **Alcohol use** significantly increased the risk of poor treatment outcomes in both DR- and MDR-TB patients. | | ↑ | Y | Y | Y | Y | Y | CT | Y | Y | Y | 6 |
| Rajendran et al., 2020 (25) |  | Malaysia | TB | Patients with TB | **Drug abuse, alcohol use** and **smoking** were strongly associated with the prevalence of MDR-TB. | | ↑ | Y | Y | Y | CT | N | N | N/A | Y | Y | 5 |
| Rumbwere Dube et al., 2018 (33) |  | Global | HIV | Not given *(adults)* | Being a **current smoker, binge drinking, alcohol misuse** and **drug use** were predictors of HIV infection. | | ↑ | Y | Y | Y | Y | Y | Y | N/A | Y | Y | 7 |
| Sales-Peres et al., 2020 (56) | ✓ | Global | COVID-19 | Patients with COVID-19 | Patients with **severe obesity** are at high risk of severe COVID-19, IMV, ICU admission and mortality, independent of age, race, sex and co-morbidities. | | ↑ | Y | Y | Y | Y | Y | Y | Y | Y | Y | 7 |
| Samokhvalov et al., 2010 (35) | ✓ | Global | Pneumonia (CAP) | Not given | **Alcohol** was a risk factor for pneumonia. | | ↑ | Y | Y | Y | CT | Y | Y | Y | Y | Y | 6 |
| Samuels et al., 2018 (34) | ✓ | Global | TB | Patients treated for MDR/XDR TB | **Alcohol misuse** was associated with unsuccessful treatment outcomes. Outcomes were similar in people that **smoked** compared to non-smokers. | | ↑- | Y | Y | Y | CT | Y | Y | Y | Y | Y | 6 |
| Sanchez-Ramirez et al., 2020 (68) | ✓ | China, USA | COVID-19 | Patients with COVID-19 | **Smoking** was associated with severe outcomes. | | ↑ | Y | Y | Y | Y | N | N | Y | Y | Y | 5 |
| Simou et al., 2018 (31) | ✓ | Global | Pneumonia (CAP) | Generally representative populations *(adults)* | **Alcohol** **consumption** increased the risk of CAP. | | ↑ | Y | Y | Y | Y | Y | Y | Y | Y | Y | 7 |
| Simou et al., 2018 (32) | ✓ | Global | Active TB | General and hospital populations *(adults)* | **Alcohol consumption** was an important risk factor for the development of TB. | | ↑ | Y | Y | Y | Y | Y | Y | Y | Y | Y | 7 |
| Sonego et al., 2015 (74) | ✓ | LMIC | Pneumonia; ALRI | Hospital and community populations *(< 5 years)* | **SHS** exposure increased the odds of death from acute lower respiratory disease, including pneumonia, in children. | | ↑ | Y | Y | Y | Y | Y | Y | Y | Y | Y | 7 |
| Song et al., 2020 (50) |  | Global | Influenza, pneumonia | Not given *(older adults)* | Prolonged moderate **aerobic exercise** may help to reduce the risk of influenza-related infection. | | ↑ | Y | Y | Y | Y | N | N | N/A | Y | Y | 5 |
| Sublette et al., 2013 (28) |  | *Not given* | HCV | Patients with chronic HCV | The effect of **alcohol** **use** on the ability to achieve an SVR depends on the amount consumed, with an adverse impact among those consuming >70g per day. The relationship between dietary intake and SVR could not be determined. | | ↑- | Y | Y | Y | CT | Y | Y | Y | Y | Y | 6 |
| Torres et al., 2019 (30) | ✓ | Global | TB | Patients treated for pulmonary TB | **Alcohol use** and **smoking** negatively affected the success of TB treatment. | | ↑ | Y | Y | P | Y | Y | Y | Y | Y | Y | 6 |
| Velloza et al., 2019 (26) | ✓ | Sub Saharan Africa | HIV | Patients receiving antiretroviral therapy *(adults)* | **Alcohol users** receiving antiretroviral therapy had greater likelihood of viral non-suppression. | | ↑ | Y | Y | Y | Y | Y | Y | Y | Y | Y | 7 |
| Waitt et al., 2011 (37) |  | Global | TB | Patients being treated for TB *(adults)* | In regions with low TB incidence and HIV prevalence, risk factors include **alcohol** and **substance misuse**. No conclusions were drawn for **smoking** but the review reported no associations with TB death. | | ↑ | Y | Y | Y | CT | N | N | N/A | Y | Y | 5 |
| Wang et al., 2018 (63) | ✓ | Global | TB | Patients with TB *(≥ 15 years)* | **Tobacco smoking** was a risk factor for DR-TB regardless of study design, smoking status, and type of DR-TB. | | ↑ | Y | Y | Y | Y | Y | Y | Y | Y | Y | 7 |
| Wang et al., 2020 (62) | ✓ | *Not given* | TB | Patients being treated for TB | **Smoking** was associated with unfavourable treatment outcomes for TB. | | ↑ | Y | Y | Y | CT | Y | Y | Y | Y | Y | 6 |
| Zhao et al., 2015 (0970) |  | China | HIV | General and selected populations | **Illicit drug use** was associated with a higher prevalence of HIV. | | ↑ | Y | Y | Y | Y | Y | Y | N/A | Y | Y | 7 |
| Zhao et al., 2020 (1164) | ✓ | China | COVID-19 | Patients with COVID-19 | **Active smoking** increased the risk of severe COVID-19, but the result was influenced by one study. After its removal, the association was non-significant. | | ↑- | Y | Y | Y | Y | Y | Y | Y | Y | Y | 7 |

Y, yes; N, no; CT, can’t tell; P, partially. * 1 = extensive flaws, 3 = major flaws, 5 = minor flaws, 7 = minimal flaws. *↑ = increased risk; ↓ = decreased risk; — = no association reported

MA = meta-analysis; TB = tuberculosis; DR-TB = drug-resistant tuberculosis; MDR-TB = multi-drug resistant tuberculosis; DS-TB = drug-susceptible tuberculosis; XDR-TB = extensively drug resistant tuberculosis; LTBI = latent tuberculosis infection; HIV = human immunodeficiency virus; AIDS = acquired immunodeficiency syndrome; HCV = hepatitis C virus; HBV = hepatitis B virus; HDV = hepatitis D virus; ARLI = acute lower respiratory infection; CAP = community acquired pneumonia; IBD = invasive bacterial disease; IPB = invasive pneumococcal disease; IMD = invasive meningococcal disease; IHD = invasive hib disease; HBsAg = Hepatitis B surface antigen; PWID = people who inject drugs; SHS = second hand smoke; ETS = environmental tobacco smoke; SVR = sustained virological response; ICU = intensive care unit; IMV = invasive mechanical ventilation; SES = socio-economic status.
