# Supplementary material for: The impact of behavioural risk factors on communicable diseases: a systematic review of reviews
Source: BMC Public Health. 2021 Nov 17;21:2110. doi: 10.1186/s12889-021-12148-y (PMC8596356; doi:10.1186/s12889-021-12148-y)
Supplement: Supplementary file 3 — Additional file 3. [file 12889_2021_12148_MOESM3_ESM.docx]

**Supplementary File 3: Included review topics**

**Break down of included reviews by risk factor and communicable disease.**

|  | Risk factors | | | | | | |
| --- | --- | --- | --- | --- | --- | --- | --- |
|  | Alcohol | Illicit drug use | Obesity | Smoking | Second-hand smoke | Poor diet | Physical inactivity |
| TB | 8 | 4 | - | 8 | 3 | - | - |
| HIV | 4 | 4 | - | 1 | - | 1 | - |
| Hepatitis C virus | 2 | 7 | - | - | - | 1 | - |
| Hepatitis B virus | - | 1 | - | - | - | - | - |
| Invasive Bacterial Disease | 1 | - | - | 1 | 1 | - | - |
| Influenza | - | - | 4 | 1 | - | - | 1 |
| Pneumonia | 2 | - | 2 | 1 | 2 | - | - |
| COVID-19 | - | - | 4 | 6 | - | - | - |
